# Expression, Purification and Characterization of the Human Cannabinoid 1 Receptor

**DOI:** 10.1038/s41598-018-19749-5

**Published:** 2018-02-13

**Authors:** Srikrishnan Mallipeddi, Nikolai Zvonok, Alexandros Makriyannis

**Affiliations:** 1 Department of Pharmaceutical Sciences, Northeastern University, Boston, MA 02115 USA; 2 Center for Drug Discovery, Northeastern University, Boston, MA 02115 USA; 3 Department of Chemistry and Chemical Biology, Northeastern University, Boston, MA 02115 USA

## Abstract

The human cannabinoid 1 receptor (hCB1) is involved in numerous physiological processes and therefore provides a wide scope of potential therapeutic opportunities to treat maladies such as obesity, cardio-metabolic disorders, substance abuse, neuropathic pain, and multiple sclerosis. Structure-based drug design using the current knowledge of the hCB1 receptor binding site is limited and requires purified active protein. Heterologous expression and purification of functional hCB1 has been the bottleneck for ligand binding structural studies using biophysical methods such as mass spectrometry, x-ray crystallography and NMR. We constructed several plasmids for in-cell or *in vitro Escherichia coli* (*E. coli*) based expression of truncated and stabilized hCB1 receptor (hΔCB1 and hΔCB1_T4L_) variants and evaluated their competency to bind the CP-55,940 ligand. MALDI-TOF MS analysis of *in vitro* expressed and purified hΔCB1_T4L_his6 variants, following trypsin digestion, generated ~80% of the receptor sequence coverage. Our data demonstrate the feasibility of a cell-free expression system as a promising part of the strategy for the elucidation of ligand binding sites of the hCB1 receptor using a “Ligand Assisted Protein Structure” (LAPS) approach.

## Introduction

The hCB1 receptor, an integral membrane protein of the GPCR superfamily, Class A subtype^1^, is found predominantly in the central nervous system (CNS) and, to a lesser extent, in the periphery^2^. The hCB1 receptors play an important role in the central and peripheral regulation of food intake, fat accumulation, lipid and glucose metabolism, pain perception, hormonal activity, thermoregulation, as well as cardiovascular, motor, cognitive, emotional, and sensory functions^3–5^. The development of specific ligands modulating these effects could have therapeutic benefits in a variety of pathological conditions including: obesity, cardio-metabolic diseases, drug dependence, pain, and neurodegeneration^6^. Rational drug design can be greatly improved by obtaining information on the ligand-binding site interactions and the receptor conformational changes. Recently, the crystal structures of the hCB1 complex with the inverse agonists AM6538 and taranabant were reported^7,8^. However, receptor binding of ligands with different structures and functional responses suggests variability in the ligand binding domains^9^. To perform structural studies using various biophysical techniques, we need to develop a suitable expression system that produces reasonable quantities of purified active human CB1 receptor.

Owing to its simplicity and scalability, the bacterial recombinant protein expression remains the most popular choice for structural biology studies. However, bacterial cells are not well suited for GPCR expression due to the lack of machinery required both for post-translational modifications and the incorporation of mammalian membrane proteins into the bacterial membranes^10^. In spite of these difficulties, a number of GPCRs, such as Adenosine 2 A receptor^11^, Cannabinoid 2 receptor^12^, Neurotensin 1 receptor^13^, Vasopressin 2 receptor^14^, thyroid stimulating hormone receptor^15^ and Serotonin 4 A receptor^16^, have been successfully expressed using bacterial expression system. However, unmodified receptors usually exhibit poor stability and rapidly become inactive aggregates. Selective protein engineering involving mutations/truncations/insertions, addition of stabilizing fusion partners, using advanced *E. coli* expression strains, and prolonged induction times at low temperature are required for efficient expression of functionally active receptors^17,18^. Alternatively, refolding inactive inclusion bodies into functional proteins^19^, adding lipid vesicles^20^ and/or the use of non-ionic detergents (DDM and CYMAL5) have also been shown to be successful^21^.

Over the past decade, cell-free expression^22^ has become a powerful tool to express various proteins with high efficiency^23^. It provides a number of key advantages over *in vivo* expression, such as easy access to the reaction during expression thus enabling easy labeling strategies for NMR studies, and eliminating complications such as large downtime, as well as cumbersome transfection, virus-amplification, cells lysis and protein extraction steps^22^. Recent developments, such as the addition of selective detergents, nano-lipid bilayers^24,25^, amphipols, proteomicelles, peptide surfactants and liposomes^26,27^^,^ have enabled the expression of membrane proteins, including GPCRs^28^. These new developments can be used for the expression of hydrophobic proteins and provide a membrane-like environment to assist in proper folding, as well as decrease the formation of aggregates and precipitation^29^.

In this paper, we report the expression of functional *N*-terminal truncated and stabilized hCB1 receptor variants in *E. coli* cells and using *in vitro* expression system in the presence of pre-formed nanodiscs to obtain proteins suitable for “Ligand Assisted Protein Structure” (LAPS)^30–33^ studies.

## Materials and Reagents

Standard laboratory chemicals were purchased from Sigma Chemical Co. (St. Louis, MO) and Fisher Scientific (Pittsburgh, PA), if not otherwise specified. Coomassie G-250 stain, Laemmli electrophoresis sample buffer, PVDF membrane, molecular weight markers and SDS-PAGE gels were from Bio-Rad (Hercules, CA). Trypsin Gold, MS grade, was purchased from Promega (Madison, WI). MembraneMax Protein Expression Kit was purchased from Invitrogen (Carlsbad, CA). The n-Dodecyl-β-D-maltoside (DDM) and 5-cyclohexyl-1-pentyl-β-D-maltoside (CYMAL5) were purchased from Anatrace (Maumee, OH). [^3^H] CP-55,940 was provided by the National Institute on Drug Abuse, National Institutes of Health (Bethesda, MD). GF-B 96-well filter assays plates for radioactive binding assays were purchased from Perkin Elmer (Waltham, MA).

## Methods

### The hCB1 expression constructs design

#### Preparation of the pET15hΔCB1his6 and pET26shΔCB1his6 plasmids:

The truncated hCB1 gene (hΔCB1, 1.1 kbp) was amplified from the hCB1 DNA template using *Pfu* DNA polymerase (Stratagene) and primers containing *Nco*I (forward) and *Xho*I (reverse) restriction sites in an Eppendorf Mastercycler (Westbury, NY). The PCR product was ligated into pET15b or pET26b expression vectors (Novogen) following digestion with *Nco*I and *Xho*I restriction enzymes (New England BioLabs). Colonies of One Shot Top10 *E. coli* cells (Invitrogen) transformed with ligation mixtures were selected on LB agar plates with antibiotic either ampicillin (Ap −100 µg/ml for pET15b) or kanamycin (Km −25 µg/ml for pET26b). After plasmid preparation (GeneJET Plasmid Miniprep Kit, Fermentas, Maryland), the presence of truncated hCB1 gene in pET15hΔCB1his6 and pET26shΔCB1his6 were confirmed by sequencing (SeqWright).

#### Construction of the pET26shΔCB1_T4L_his6 plasmid:

a. Preparation of the T4 lysozyme DNA by PCR: The T4 lysozyme DNA was amplified from the T4 lysozyme DNA template using primers designed for overlap extension PCR cloning: Forward - **GTATATTCTCT;GGAAGGCTCACAGCCAC***AATATATTTGAAATGTTACG* and Reverse - *AACCTAATGTCCATGCGGGCTTGGTC***ATACGCGTCCCAAGTGCC** primers contained the sequence complemented to CB1 and T4 lysozyme DNA (in bold and in italic, respectively). The PCR reaction mixture was prepared with the following component: pFastbacCB2_T4L_ template (10 ng/µl), mixture of Forward and Reverse primers (100 nM each), dNTPs (200 μM each), Advantage 2 polymerase (0.5 µl) (Clontech, Mountain View, CA) in 1x Advantage 2 buffer (50 µl). Amplification cycles were carried out using a MyCycler Thermal cycler (Bio-Rad, Hercules, CA) as follows: a single denaturation step of 94 °C for 2 min was followed by 25 cycles of 94 °C for 10 s, 50 °C for 33 s and 72 °C for 1 min 33 s and completed with a final extension step of 72 °C for 10 min. The quality and quantity of the PCR product, before and after purification using the GeneJET PCR Purification (Mini) Kit, was evaluated by electrophoresis in 0.8% agarose gel.

b. Insertion of T4 lysozyme DNA into pET26shΔCB1his6: The reaction mixture contained pET26shΔCB1his6 plasmid DNA template (5 ng/µl), dNTPs (350 μM of each), T4 lysozyme PCR DNA (7.5 ng/µl, megaprimers), and Advantage 2 polymerase (0.4 µl) in Advantage 2 buffer (20 µl). The following protocol was used for linear extension of megaprimers: a single denaturation step of 95 °C for 2 min was followed by 20 cycles of 94 °C for 10 s, 50 °C for 33 s and 68 °C for 7 min and completed with a final extension step of 68 °C for 10 min. To digest pET26shΔCB1his6 DNA template, 10 µl of the reaction was mixed with 10 µl of 1x Advantage 2 buffer containing 3 U of *Dpn*I restriction enzyme (Agilent Technologies) and incubated for 2.5 h at 37 °C.

*Dpn*I digest (1 μl) was transformed into XL1-blue competent cells (Agilent Technologies, La Jolla, CA) and colonies were selected on LB agar plates containing Km. The plasmid DNA from selected colonies was purified using the GeneJET™ Plasmid Miniprep and used as template for amplification of modified the shΔCB1his6 gene with Forward and Reverse T7 primers. The PCR products were purified by the GeneJET™ PCR Purification (Mini) Kit and sequenced by SeqWright DNA technology services (Houston, Texas) to confirm the correct insertion of the T4 lysozyme and the absence of unwanted mutations.

#### Construction of the plasmid for FlaghΔCB1_T4L_his6 expression:

a. Restriction digestion and dephosphorylation of the pET26shΔCB1T4Lhis6: The plasmid pET26shΔCB1_T4L_his6 (3.0 µg) was digested with *Nde*I and *Nco*I restriction enzymes (Fermentas, Maryland), dephosphorylated by Antarctic phosphatase (New England Biolabs) and purified using the Wizard gel and PCR clean-up system (Promega, Madison, WI).

b. Phosphorylation of oligonucleotides and insertion into dephosphorylated pET26shΔCB1_T4L_his6/*Nde*I + *Nco*I plasmid: The complimentary oligonucleotides, FwhCB1flag −5′-TATGGATTATAAAGATGACGATGACAAAGC and RvhCB1flag −5′-CATGGCTTTGTCATCGTCATCTTTATAATCCA, which code for a FLAG-tag flanked with *Nde*I and *Nco*I cohesive ends, were phosphorylated separately using T4 polynucleotide kinase (Fermentas, Maryland). Purified, digested and dephosphorylated pET26shΔCB1_T4L_his6 plasmid DNA (0.05 pmol/µl) and a mixture of the phosphorylated oligonucleotides coding a FLAG-tag (0.67 pmol/µl of each) were ligated using T4 DNA ligase (New England Biolabs) at room temperature for 2 h prior to use in transformation.

c. Analysis and purification of the pET26FlaghΔCB1_T4L_his6 plasmid: The DNA ligation mixture was transformed into XL1-blue competent cells and the colonies selected on LB agar plates with Km were analyzed using PCR and DNA sequencing, with the protocol mentioned previously, to confirm the presence of oligonucleotide insertion in pET26FlaghΔCB1_T4L_his6 plasmid. Phenol extraction was used to remove traces of RNases from purified plasmid DNA.

### Expression and purification of the hCB1 receptor variants from *E. coli* cells

#### Expression of the hΔCB1his6, shΔCB1his6, shΔCB1_T4L_his6 or FlaghΔCB1_T4L_his6 proteins:

A single colony of *BL*21(DE3) cells containing either pET15hΔCB1his6 or pET26shΔCB1his6 or pET26shΔCB1_T4L_his6 or pET26FlaghΔCB1_T4L_his6 plasmid was inoculated in 5 ml LB media (containing either Ap 100 μg/ml or Km 25 μg/ml, respectively) and incubated overnight at 37 °C. Overnight culture was added to 500 ml of LB media (containing either Ap or Km, respectively) and allowed to grow overnight at 33 °C. The receptor expression was induced at OD_600_ ~3 with IPTG (final concentration 0.3 mM) and continued for 4 h at 25 °C. The cells were harvested by centrifuging at 5000 g for 15 min and stored at −80 °C.

#### Preparation of *E. coli* membranes:

Membranes from *E. coli* cells were prepared using previously published protocols^34^. Briefly, the *E. coli* cell pellet (2 g) was washed twice with 0.1 M Tris-HCl, pH 8.0 (20 ml) and resuspended in cold 0.1 M Tris-HCl containing 20% sucrose (30 ml). To the cell suspension, Halt protease inhibitor cocktail (Fisher Scientific, Pittsburgh, PA) was added (final concentration of 10 µl/ml), incubated at 37 °C for 15 min, followed by lysozyme treatment (final concentration of 0.1 mg/ml) and incubation for 15 min at 37 °C with mild agitation. To this mixture, ethylenediaminetetraacetic acid (EDTA) was added (final concentration of 10 mM) and incubated further for 10 min at 37 °C with mild agitation. The mixture was centrifuged at 12000 *g* for 20 min and washed with 0.1 M Tris-HCl containing 20% sucrose (20 ml). The pellet was then resuspended in ice-cold water (2 ml) and briefly sonicated. To this suspension, Halt protease inhibitor cocktail (final concentration of 10 µl/ml), DNAse I (1000 U), MgCl_2_ (final concentration of 1 mM) and Tris-HCl (final concentration of 50 mM) were added, briefly sonicated and incubated on ice for 1 h. This suspension was centrifuged at 100,000 g for 1 h and the membrane pellet was washed with 50 mM Tris-HCl (20 ml) and centrifuged again at 100,000 g for 1 h. The membrane pellet was resuspended in 10 ml of 50 mM Tris-HCl and stored at −80 °C in aliquots. Membrane protein was quantified with a Bradford dye-binding method (Bio-Rad Laboratories).

#### Saturation binding assay with membrane preparations of the hΔCB1 receptor variants:

The 96-well GF/B filtration plates (Perkin Elmer) were pre-treated with 0.5% polyethylenimine (PEI) for 3 h at 4 °C. The plates were placed on a vacuum manifold (Pall Corporation) and washed twice with 200 μl of binding buffer (BB −25 mM Tris-HCl, 5 mM MgCl_2_, 1 mM EDTA, 0.1% BSA, pH 7.4). Saturation binding assays used 25 μg of protein in each assay well. The [^3^H] CP-55,940 was diluted in BB to ligand concentrations ranging from 0.039 to 40 nM. Nonspecific binding was assayed in the presence of unlabeled CP-55,940 (2 μM). The reaction was incubated at 30 °C for 1 h with gentle agitation. The resultant material was transferred to the pre-treated Unifilter GF/B filter plate using a Packard Filtermate-196 Cell Harvester (Perkin Elmer). Filter plate was washed four times with ice-cold wash buffer (50 mM Tris-HCl, 5 mM MgCl_2_, 0.5% BSA, pH 7.4) to remove any unbound ligand. Scintillation fluid (40 μl/well, Microscint 20 from Perkin Elmer) was added to each well and the plates were counted using TopCount NXT™ Microplate Scintillation and Luminescence Counter (Perkin Elmer). The data obtained was processed using Microsoft Excel and Prism 5 (GraphPad, La Jolla, CA). All concentration points were performed in triplicate and data points used for plotting are baseline corrected. *B*_max_ and *K*_d_ values were calculated by nonlinear regression using Graphpad Prism version 5.03 (one site-binding analysis equation *Y* = *B*_max_ × *X*/(*K*_d_ + *X*)) on a Windows platform.

#### IMAC purification of his6-tagged the hΔCB1 receptor variants:

The his-tagged hΔCB1 receptor variants were extracted from either *E. coli* cells or membrane preparations and purified using Talon IMAC resin (Clontech). Cell pellet (200 mg) or membrane fraction (10 mg) was resuspended in 2 ml or 1.2 ml, respectively, purification buffer (PB, 50 mM sodium phosphate, 300 mM NaCl, 10% glycerol, pH 7.4) with 1% DDM and lysed on ice by two 33 s sonication cycles, each cycle consisting of a 1 s burst at 50 W separated by a 5 s interval. The lysate was centrifuged at 16000 × g for 15 min and the resulting supernatant was collected, diluted with an equal volume of PB, added to Talon metal affinity resin (400 μl) pre-equilibrated in PB with 0.5% DDM and incubated for 2 h at 4 °C on a rotating wheel. The resin was washed twice with PB containing 0.2% DDM (2 × 1.0 ml) and the protein was eluted 5 times using PB containing 0.2% DDM and 250 mM imidazole (5 × 100 μl). SDS-PAGE and western blotting analysis were performed on the aliquot of samples taken during purification according to the procedure as detailed below.

#### Anti-FLAG immunoaffinity purification of the FlaghΔCB1_T4L_his6:

The cell pellet (200 mg) was suspended in PB (2 ml) with 1% DDM and lysed on ice by three 33 s sonication cycles; each cycle consisted of a 1 s burst at 50 W separated by a 5 s interval. The lysate was centrifuged at 16000 × g for 15 min and the resulting supernatant was collected, diluted with an equal volume of PB, added to ANTI-FLAG M2 affinity gel (200 μl) (Sigma) pre-equilibrated in PB with 0.5% DDM, and incubated for 2 h at 4 °C on a rotating wheel. The resin was washed twice with PB containing 0.2% DDM, pH 7.4 (2 × 200 μl) and the protein was eluted 5 times using PB containing 0.2% DDM and 150 μg/ml of FLAG peptide (5 × 100 μl) (Sigma). SDS-PAGE and western blotting analysis were performed on the aliquot of samples taken during purification according to the procedure as described below.

### Cell-free expression and purification of the shΔCB1_T4L_his6 or FlaghΔCB1_T4L_his6 variants

#### *In vitro* expression reaction:

The cell-free MembraneMax Protein Expression Kit (Invitrogen, Carlsbad, CA) and the pET26shΔCB1_T4L_his6 or pET26FlaghΔCB1_T4L_his6 plasmid DNA were used for expression of CB1 receptor variants according to the manufacturer’s recommended protocol with minor modifications. The *in vitro* expression reaction, containing plasmid DNA (1 μg), and Porcine Optizyme RNase Inhibitor (400 units) (Fisher Scientific, Pittsburgh, PA) in reaction buffer mixture (final volume of 100 μl), was mixed at 300 rpm on a plate shaker for 30 min at 30 °C. Then 100 μl of feed buffer mixture was added (final reaction volume of 200 μl) and the incubation continued for another 90 min according to the manufacturer’s recommendation. The expression of hΔCB1_T4L_his6 protein was confirmed by SDS-PAGE analysis in AnykD Mini-PROTEAN^®^ TGX™ precast polyacrylamide gels (Bio-Rad), followed by western blot analysis using his-tag based immuno-detection as detailed below. The total protein concentration was determined using Quick Start™ Bradford Protein Assay kit (Bio-Rad) according to the manufacturer’s recommended protocol and the absorbance values were measured at 595 nm using the EnVision™ Multilabel Plate Reader (Perkin Elmer, Waltham MA).

#### Purification of the *in vitro* expressed hCB1 receptor:

a. Immobilized metal affinity chromatography (IMAC) purification of the solubilized shΔCB1_T4L_his6 receptor or nanodisc-shΔCB1_T4L_his6 receptor complex: The *in vitro* expressed receptor was purified either as solubilized shΔCB1_T4L_his6 protein or as a nanodisc-shΔCB1_T4L_his6 complex. To purify the solubilized receptor, shΔCB1_T4L_his6 *in vitro* expression reactions (200 μl) were diluted with 2 volumes of PB (pH 8.0) containing 1% DDM and incubated for 1 h at 4 °C on a rotating wheel. The samples were then added to PB (pH 8.0) equilibrated Talon resin (100 μl) (Clontech) and incubated for 2 h at 4 °C on a rotating wheel. The resin was washed twice with PB (pH 8.0) (2 × 500 μl) and the protein was eluted 5 times using 150 mM Imidazole in PB (pH 8.0) (5 × 50 μl). Sample aliquotes taken during purification were analyzed by western blot analysis according to the procedure given below.

The nanodisc-shΔCB1_T4L_his6 receptor complex was purified directly from the *in vitro* expression reaction using Talon (IMAC) resin in PB (pH 8.0) without detergent.

b. Anti-FLAG immunoaffinity purification of the nanodisc-FlaghΔCB1_T4L_his6 receptor complex: The *in vitro* expression reaction of FlaghΔCB1_T4L_his6 (200 μl) was diluted with the PB (pH 8.0) (200 μl), added to ANTI-FLAG M2 affinity gel (100 μl) (Sigma, St. Louis, MO) equilibrated in PB (pH 8.0) and incubated for 2 h at 4 °C on a rotating wheel. The resin was washed twice with PB (2 × 100 μl) and the protein was eluted using PB containing FLAG peptide (150 μg/ml, Sigma) (5 × 100 μl). Sample aliquotes taken during purification were analyzed by western blot according to the procedure given below.

#### Saturation binding of [^3^H] CP-55,940 to the nanodisc-hΔCB1_T4L_his6 receptor complex:

The radioactive binding assays were performed with [^3^H] CP-55,940 radioligand. GF/B filtration plates (96-wells; Perkin Elmer) were pre-treated with 0.5% polyethylenimine (PEI) for 3 h at 4 °C. The plates were placed on a vacuum manifold (Pall Corporation, Port Washington, NY) and washed twice with rinsing buffer (RB −50 mM Tris-HCl, 150 mM NaCl, pH 7.4) (2 × 200 μl). The radioligand-receptor binding assay was performed with six radioligand concentrations (1–50 nM) with 60 μg protein in RB with 0.1% BSA (60 μl final volume). Non-specific binding was determined in wells containing an excess of cold CP-55,940 (2 μM). After 1 h incubation at 30 °C, the samples were transferred in triplicates, to selected wells of a PEI pre-treated, 96-well GF/B filtration plate. The plate was then placed on a vacuum manifold and washed with RB containing 0.1% BSA under 25 mm Hg vacuum. Microscint 20 scintillation fluid (40 μl, Perkin Elmer) was added to each well and the plates were counted using a TopCount NXT Microplate Scintillation and Luminescence Counter (Perkin Elmer). All concentration points were performed in triplicate and the data points used for plotting were baseline corrected. The *B*_max_ and *K*_d_ values were calculated via nonlinear regression using Graphpad Prism version 5.03 (one site-binding analysis equation *Y* = *B*_max_ × *X*/(*K*_d_ + *X*)).

### Western blotting

Proteins in each sample were separated using AnykD Mini-PROTEAN TGX precast polyacrylamide gels at 150 V for 10 min followed by 200 V for 30 min. The blotting transfer to polyvinylidene fluoride (PVDF) membranes (Bio-Rad) was performed in the Trans-*Blot* SD *semi*-*dry* electrophoretic transfer cell (Bio-Rad) using Towbin buffer (25 mM Tris, 192 mM glycine, 10% methanol and 0.1% SDS) for 10 min at 10 V followed by for 20 min at 15 V. The PVDF membrane was washed using 1× TBS with 0.25% Tween-20 twice for 10 min, incubated in blocking buffer (Qiagen, Valencia, CA**)** for 1 h, washed twice using 1× TBS with 0.25% Tween-20 and 0.2% Triton, followed by another wash using 1× TBS with 0.25% Tween-20 for 10 min. The membrane was incubated with the Penta-His antibody horseradish peroxidase conjugate (Qiagen, Germantown, MD) in blocking buffer for 1 h on a gel rocker. The washing steps were repeated and the proteins were visualized using an ECL Western Blotting Analysis System (GE Healthcare, Piscataway, NJ). The image was captured using a FluorChem SP Imaging System (Alpha Innotech Santa Clara, CA).

### Mass spectrometric analysis of the purified CB1 receptor

#### Reduction, alkylation and in-solution trypsin digestion of the purified CB1 receptor:

The samples for MS analysis were prepared using the previously reported procedures^32^. Briefly, the purified CB1 receptor in PB (35 μl) was reduced using dithiothreitol (DTT, 20 mM) then alkylated using iodoacetamide (IAM, 50 mM); each of these incubations was at RT for 1 h. The mixture was then desalted with 25 mM ammonium bicarbonate containing 0.05% CYMAL5 using Micro BioSpin 6 columns (Bio-Rad). The samples were subjected to overnight digestion with MS-grade Tryspin Gold (Promega, Madison, WI) at 37 °C. The digests were analyzed immediately or stored at −80 °C until further processing.

Alternatively, the tryptic peptide mixture was concentrated using Zip-tip-based extraction via the manufacturer recommended protocol. Briefly, the C4 Zip-tip was wetted with 50% Acetonitrile (ACN) and equilibrated with 0.1% Trifluoroacetic acid (TFA). The peptide mixture was bound to the equilibrated Zip-tip, washed with 0.1% TFA, and eluted with 95% ACN in 0.1% TFA.

#### MALDI-TOF/TOF analysis of the FlaghΔCB1his6 receptor tryptic peptides:

The tryptic peptide samples were analyzed, either directly or following Zip-tip-based extraction, on a MALDI TOF/TOF 4800 (AB SCIEX) instrument in both reflectron and linear modes. All MS spectra were externally calibrated using a mixture of peptide standards [des-Arg1-bradykinin at *m/z* 904.4681; angiotensin I at *m/z* 1296.6853; Glufibrino peptide at *m/z* 1570.6774; ACTH (clip 1–17) at *m/z* 2093.0867; ACTH (clip 18–39) at *m/z* 2465.1989; and ACTH (clip 7–38) at *m/z* 3657.9294]. The instrument was calibrated in MS/MS mode using five daughter ions (*m/z* 175.119, 684.346, 813.389, 1056.475 and 1441.634) generated from the fragmentation of the Glu-fibrino peptide (*m/z* 1570.6774.) MS/MS spectra were acquired on selected ions of interest under the following conditions: precursor isolation resolution of 200; collision energy of 2 kV; cell pressure of 2 × 10^−5^ torr; air as collision gas. All spectral data points were accumulated following analysis in multiple locations on each sample spot. The theoretical molecular weights of expected peptides following reduction, alkylation and trypsin digestion were calculated using MS Digest (UCSF MS facility, San Francisco, CA). The MS spectra were then analyzed by comparing the monoisotopic *m/z* values obtained from MALDI-MS analysis with the theoretical molecular weights using FindPept software (Swiss Institute of Bioinformatics, Geneva, Switzerland).

### Data Availability

No datasets were generated or analyzed during the current study.

## Results and Discussion

### Bacterial expression of the truncated CB1 receptor

**Figure 1:**
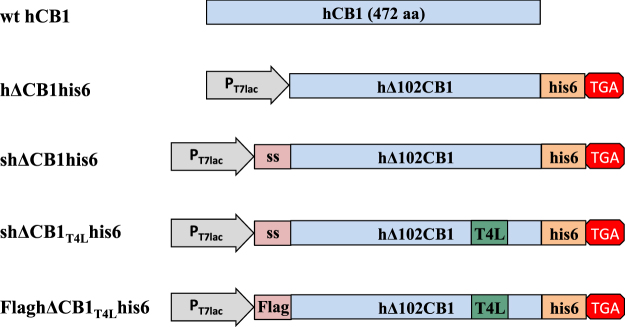
Schematic diagrams of the wt hCB1 protein and the different constructs used in *E. coli* cells and cell-free expression of the hΔCB1his6 variants.

We had previously expressed the hCB1 receptor with an *N*-terminal 102 amino acid truncated residues (hΔCB1) in *HEK*293 cells and found that truncation does not affect the binding of CP-55,940, a potent CB1 agonist (unpublished results). The DNA encoding the hΔCB1 with hexa-histidine tag at the *C*-terminus (hΔCB1his6) was cloned into pET15b expression vector to form pET15hΔCB1his6 construct that would produce the hΔCB1his6 receptor. To facilitate receptor insertion into *E. coli* membranes, another pET26shΔCB1his6 construct, encoding the shΔCB1his6 receptor with a pelB signal sequence (s - MKYLLPTAAAGLLLLAAQPAMA) at the *N*-terminus and a hexa-histidine tag at the C-terminus was constructed (Fig. 1).

The expression of the hΔCB1his6 and shΔCB1his6 receptors was investigated in the *BL*21(DE3) *E. coli* strain transformed with pET15hΔCB1his6 or pET26shΔCB1his6 plasmids after 3–5 h induction with 0.3 mM IPTG at 27 °C (Supplementary Fig. S1). Saturation binding experiments with [^3^H] CP-55,940 and fresh spheroplast-based *E. coli* membrane preparations generated saturation curves with a *B*_max_ of 1100 pmol/g (am^N^, *K*_d_ ~6.7 nM) and 1500 pmol/g (bm^N^, *K*_d_ ~5.7 nM) for hΔCB1his6 and shΔCB1his6, respectively (Supplementary Fig. S2). The presence of *E. coli* signal sequence preceding the hΔCB1 improved the receptor insertion efficiency into the membranes (*B*_max_ of 1500 versus 1100 pmol/g). However, western blot analysis of the hΔCB1his6 and shΔCB1his6 membrane preparations, both fresh (am^N^) and after overnight storage at −80 °C (am^O^ and bm^O^), demonstrated a significantly greater receptor content in the fresh samples, suggesting that the receptor membrane preparations are unstable during storage (Supplementary Fig. S1).

Furthermore, the quality and quantity of the hΔCB1his6 or shΔCB1his6 proteins purified by immobilized metal affinity chromatography (IMAC) directly from *E. coli* cell lysate or after membrane preparation and solubilization were insufficient for comprehensive mass spectrometric and structural studies. The causes of this problem are due to the receptor instability and/or aggregation during purification, as well as the presence of significant quantities of co-purified *E. coli* proteins in the eluates (data not presented).

To address stability and purification issues of the expressed hCB1 receptor, we performed two major modifications in the pET26shΔCB1his6 construct. Previously, it was shown that incorporation of the T4 Lysozyme into the 3rd intra-cellular loop (ICL3) of a GPCR improved receptor stability^35^. To introduce the T4 Lysozyme into the ICL3 of the shΔCB1his6 receptor, an overlap extension method was used. We designed the T4 Lysozyme PCR oligonucleotide primers with addition to each at 5′-end short sequences complemented to the CB1 insertion/substitution position. After amplification, the T4 Lysozyme DNA, flanked with CB1 complimentary ends, was purified and used as megaprimers for the overlap extension procedure (Supplementary Fig. S3). Furthermore, we introduced a Flag-tag at the *N*-terminus of the hCB1 protein for immunoaffinity chromatography purification using ANTI-FLAG M2 resin. A pelB DNA fragment in pET26sΔhCB1_T4L_his6 plasmid located between *Nde*I and *Nco*I restriction sites was replaced with a short oligonucleotide duplex flanked with the same restriction sites, coding in-frame with receptor a Flag-tag sequence (DYKDDDDK). A final pET26FlaghΔCB1_T4L_his6 construct, encoding the truncated hΔCB1 receptor with an *N*-Flag tag and *C*-his6 tag and stabilized by T4 Lysozyme, was generated.

The expression of the FlaghΔCB1_T4L_his6 receptor was conducted in *E. coli* strains, *BL*21(DE3) and Rosetta-gami 2(DE3)pLysS, under different conditions including: variation in cell density, IPTG concentration, induction time and temperature. The soluble versus insoluble (inclusion bodies) receptor expression was evaluated after 3, 6 and 9 h at 25 °C using anti-his western blot analysis. The amount of detergent-solubilized receptor remained constant after 3, 6 and 9 h induction, suggesting that initially expressed receptor was stabilized by insertion into *E. coli* membrane. However, after membrane saturation (3–4 h), the majority of the receptor accumulated in cytoplasm was aggregated, forming insoluble inclusion bodies (Supplementary Fig. S4). We determined that expression of the FlaghΔCB1_T4L_his6 receptor in *BL*21(DE3) *E. coli* cells, with a 4 h incubation at 25 °C following induction with 0.4 mM IPTG, provided the best receptor expression profile (highest ratio of detergent solubilized receptor to receptor in inclusion bodies). Interestingly, the expression of FlaghΔCB1_T4L_his6 receptor in Rosetta-gami 2 (DE3)pLysS *E. coli* cells, under similar conditions, mostly produced detergent insoluble protein (95%, data not presented).

**Figure 2:**
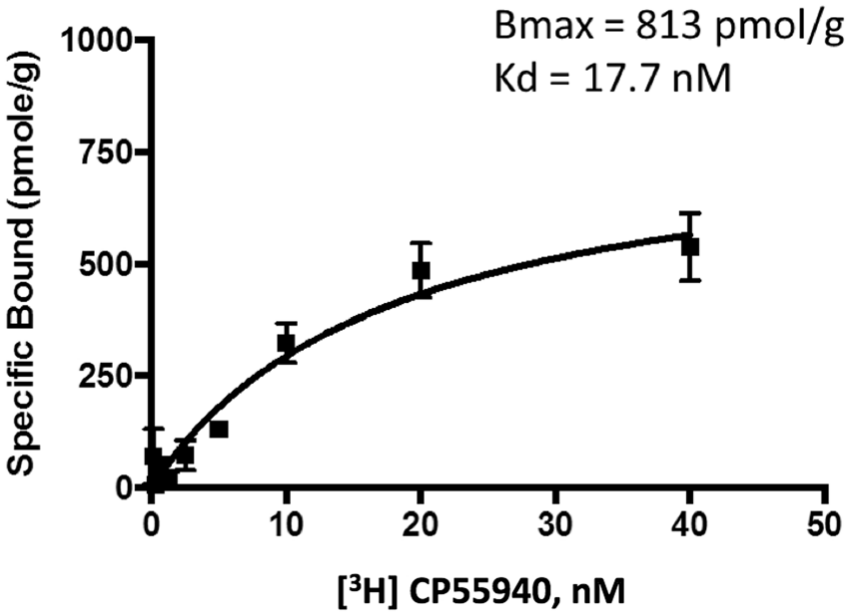
[^3^H] CP-55,940 saturation binding to the FlaghΔCB1_T4L_his6 receptor membrane preparation from *E. coli* cells. The 6-point binding assay was performed in PEI pre-treated 96-well GF/B filtration plates. The plates were counted using a TopCount NXT Microplate Scintillation and Luminescence Counter. The data obtained was processed using GraphPad Prism 5, as detailed above.

The saturation binding experiment in PEI pre-treated GF/B filtration plates with the cannabinergic radioligand [^3^H] CP-55,940 and the spheroplast-based *E. coli* membrane preparation generated a saturation curve with a *B*_max_ of 813 pmol/g and a *K*_d_ of 17.7 nM (Fig. 2). The PEI pre-treatment was expected to decrease the non-specific binding of the receptors to the filter plates. However, it should be noted that non-specific [^3^H] CP-55,940 binding in the presence of CP-55,940 (2 μM) constituted about 55% of the total binding. This high non-specific binding has been attributed to the presence of a large number of non-specific interactions with the bacterial membrane and was also observed with the human CB2 receptor expressed in *E. coli*^34^. Replacement of the signal sequence by FLAG-tag reduced the *B*_max_ of the hΔCB1 receptor in the membrane fraction (813 versus 1500 pmol/g) and increased the receptor content in the inclusion bodies (Supplementary Fig. S4).

**Figure 3:**
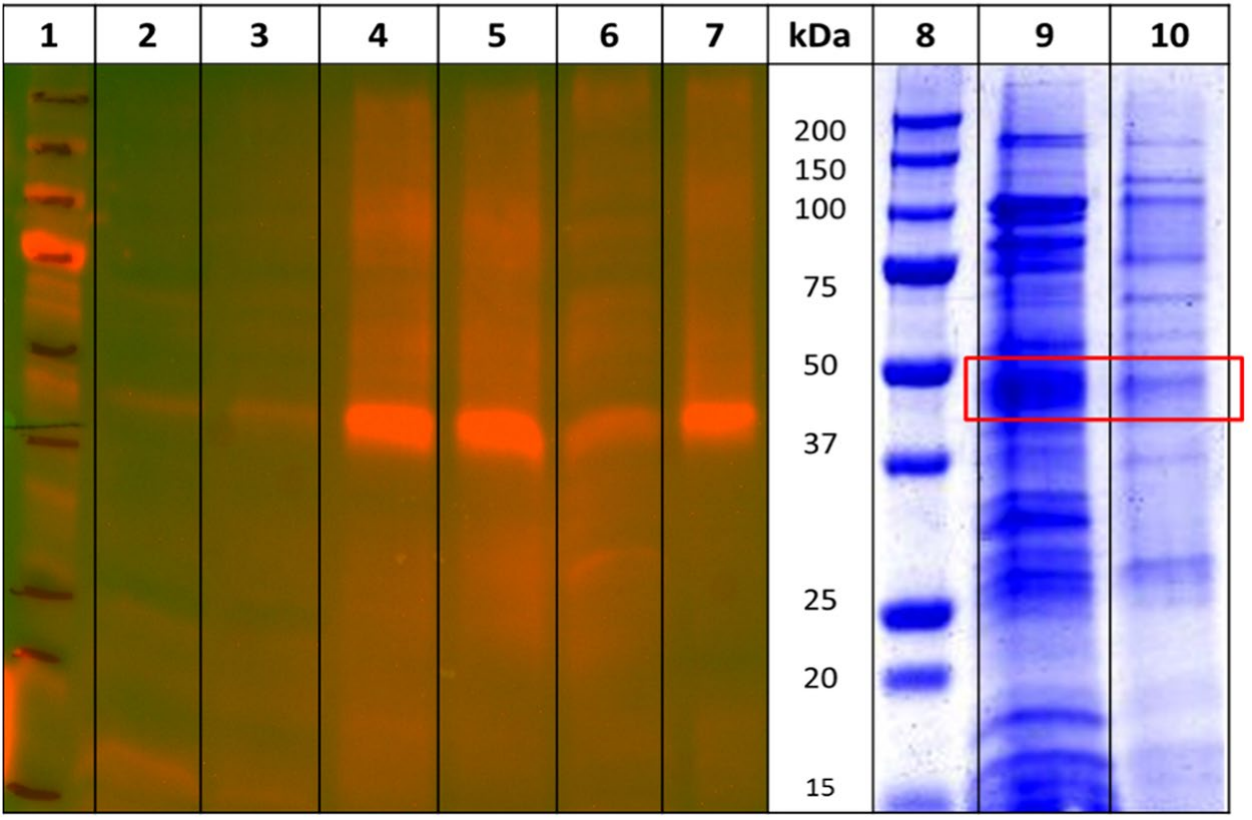
Anti-His western blot (lanes 1–7) and coomassie-stained SDS-PAGE (lanes 8–10) analysis of FlaghΔCB1_T4L_his6 receptor affinity purification. The lane contents are as follows: proteins in cells lysate unbound to FLAG M2 affinity resin (2), resin wash before (3) and after elution (6), FlagΔhCB1_T4L_his6 receptor eluates from FLAG M2 affinity resin (4, 5, 10) and FlagΔhCB1_T4L_his6 receptor eluates from His-tag based IMAC Talon resin (7, 9). The red box denotes the bands corresponding to the FlaghΔCB1_T4L_his6 receptor.

The *BL*21(DE3) *E. coli* cells, containing the recombinant FlaghΔCB1_T4L_his6 receptor, were resuspended in solubilization buffer and subjected to mild sonication. The solubilized proteins were separated from the insoluble inclusion bodies and cell debris by high-speed centrifugation. The purification of FlaghΔCB1_T4L_his6 receptor was performed by either immobilized metal affinity chromatography on a Talon resin or immunoaffinity chromatography on an ANTI-FLAG M2 affinity gel, using the previously described procedures. The eluates from the two purification methods were analyzed using anti-His western blotting and coomassie staining of SDS-PAGE gel (Fig. 3). The expected bands corresponding to the FlaghΔCB1_T4L_his6 receptor were observed in samples eluted from both resins. However, we did not perform mass spectrometric characterization studies due to the overall poor protein yields (~10 μg of the FlaghΔCB1_T4L_his6 receptor from 1 L of media) and purity (20–30%).

### Cell-free expression of truncated CB1 receptor

The current GPCR cell-based expression systems are time consuming and expensive for producing pure, functional and stable receptors suitable for structural studies such as X-ray crystallography, NMR, etc. Cell-free expression systems provide the quickest connection between vector construction and protein expression that might accelerate receptor expression, thus overcoming some of the previously mentioned difficulties. The presence of the T7 promoter in all the different hΔCB1 constructs used in *E. coli* cell expression (Fig. 1) allowed us to evaluate their potentials for *in vitro* expression experiments.

**Figure 4:**
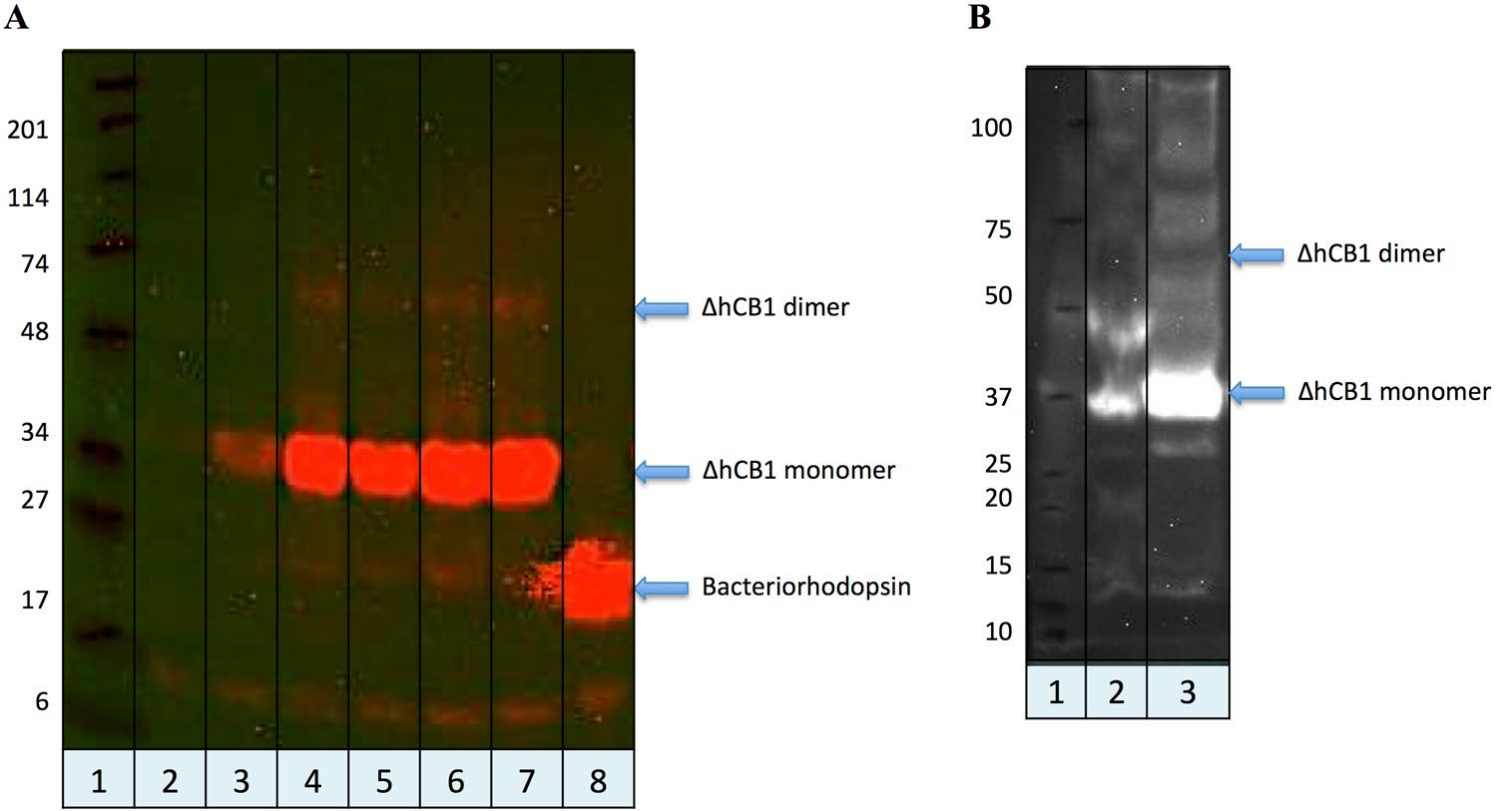
(**A**) Analysis of the hΔCB1his6 cell free expression optimization using anti-His western blot. Lane 2, 3, 4 represent the *in vitro* expression containing 0.65, 2 and 4 u/μl of Optizyme RNase inhibitor in the reaction, respectively; Lane 5, 6, 7 depicts the *in vitro* hΔCB1 expression from 5, 10 and 20 ng/μl of plasmid DNA (pET15hΔCB1his6) respectively; Lane 8 is the Bacteriorhodopsin positive control. (**B**) Anti-His western blot analysis of the cell-free expression of shΔCB1_T4L_his6 (line 2) and FlaghΔCB1T4Lhis6 (line 3) receptors. Lane 1 - Protein size markers.

The cell-free expression of the truncated hCB1 receptor variants was conducted based on commercially available MembraneMax protein expression kit (Invitrogen). The initial trials of *in vitro* hCB1 expression were performed with the DNA of truncated hCB1 gene, which was generated by PCR using T7 promoter and terminator primers on pET15hΔCB1his6 or pET26shΔCB1his6 templates (Fig. 1). The *in vitro* expression of the hΔCB1 or shΔCB1 protein from PCR DNA was not detected by western blot analysis. When the RNase free plasmid pET15hΔCB1his6 or pET26shΔCB1his6 DNA was used for cell-free expression with manufacturer’s recommended protocol, the hΔCB1his6 protein was observed at 37 kDa and 70 kDa corresponding to the monomer and dimer receptor, respectively.

Optimization of the *in vitro* hΔCB1his6 protein expression conditions was conducted with the addition of ribonuclease inhibitor (Optizyme RNase inhibitor; 0.65, 2 and 4 u/μl), using various concentrations of template DNA (5, 10 and 20 ng/μl) and extended incubation time (from 2 to 4 h). The protein yield was significantly increased upon the addition of Optizyme RNase inhibitor (4 u/μl) to the cell-free expression reaction mixture (Fig. 4). The yield of the *in vitro* expressed hΔCB1his6 protein for reactions containing 10 or 20 ng/μl of plasmid DNA were similar; however, the yield was lower when the template concentration was 5 ng/μl. At a template concentration of ~10 ng/μl, the *in vitro* reaction components were completely involved, leading to protein production saturation; adding greater amounts of the DNA template unable to provide higher protein yield. Moreover, extending the reaction incubation time to 4 h did not increase the yield of the receptor (Fig. 4A). The best expression was observed with a plasmid DNA and RNase inhibitor concentration of 10 ng/μl and 4 units/μl, respectively, and an incubation time of 2 h with feed buffer addition after the first 30 min. Using the optimal conditions for both pET15hΔCB1his6 and pET26shΔCB1his6 constructs, a considerable amount of receptor was expressed *in vitro* and observed by western blot analysis at 37 kDa (Fig. 4B). However, the saturation binding assays (both filtration and size-exclusion methods) showed that the expressed hΔCB1 receptor did not show any specific binding to [^3^H] CP-55,940, a standard cannabinoid receptor ligand. It was therefore concluded that the expressed protein was functionally inactive, which may be due to either improper folding during expression or instability of the receptor associated with nanodiscs in assays.

**Figure 5:**
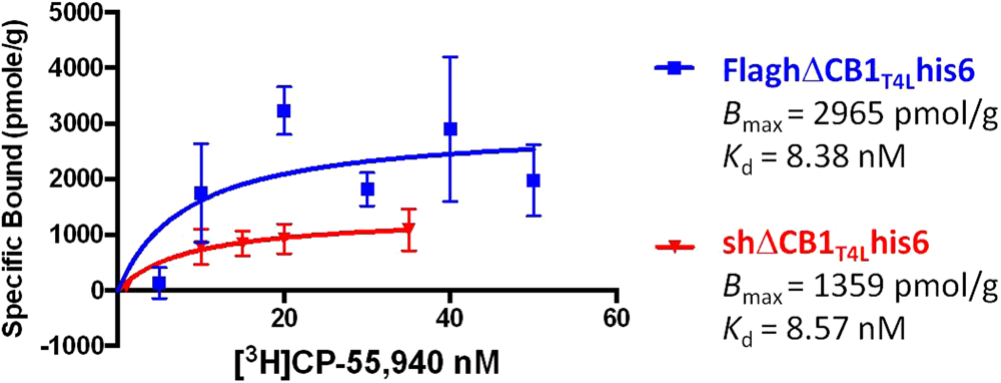
[^3^H] CP-55,940 saturation binding to the cell-free expressed shΔCB1_T4L_his6 and FlaghΔCB1_T4L_his6 receptors. The 6-point radioligand-binding assay was performed in PEI pre-treated GF/B filtration plates and the analyzed using GraphPad Prism 5.

The shΔCB1_T4L_his6 and FlaghΔCB1_T4L_his6 receptor variants, stabilized by T4 Lysozyme incorporation into the 3rd ICL, were expressed *in vitro* using optimized conditions. Interestingly, the FlaghΔCB1_T4L_his6 was expressed with a significantly higher yield than shΔCB1_T4L_his6 protein, as was determined by the western blot (Fig. 4B). The PEI pre-treated GF/B filtration plates were used in the [^3^H] CP-55,940 binding assays with *in vitro* expression reactions containing the shΔCB1_T4L_his6 or FlaghΔCB1_T4L_his6 receptor-nanodisc complex to increase the specific/nonspecific radioligand binding ratio. To determine the non-specific radioligand binding, an excess of cold CP-55,940 compared to [^3^H] CP-55,940 (2 μM and 0.5 nM, respectively) was used. Saturation binding experiments, using [^3^H] CP-55,940, produced curves (Fig. 5) with *B*_max_ of 1359 pmol/g and 2965 pmol/g and *K*_d_ of 8.57 nM and 8.38 nM for the shΔCB1_T4L_his6 and FlaghΔCB1_T4L_his6 receptor-nanodisc complexes, respectively. This confirmed that the expressed both shΔCB1_T4L_his6 and FlaghΔCB1_T4L_his6 receptors were functionally active with respect to ligand binding.

We used IMAC on TALON resin to purify the shΔCB1_T4L_his6 receptor or receptor-nanodisc complex. IMAC purification, following DDM extraction of the shΔCB1_T4L_his6 from the nanodisc complex, resulted in less than 50% protein recovery (data not presented). This poor purification yield was attributed to the aggregation and precipitation of the receptor during the transition of the shΔCB1_T4L_his6 from a stable complex with nanodiscs to the less stable complex with detergent micelles. Therefore, the nanodics-shΔCB1_T4L_his6 complex was directly purified from the *in vitro* reaction using IMAC and evaluated by western blot analysis. The purified receptor constituted about 60–70% of the total *in vitro* expressed shΔCB1_T4L_his6 protein. However, coomassie-stained SDS-PAGE analysis revealed that the purity of the shΔCB1_T4L_his6 protein was actually less than 50% (data not presented).

**Figure 6:**
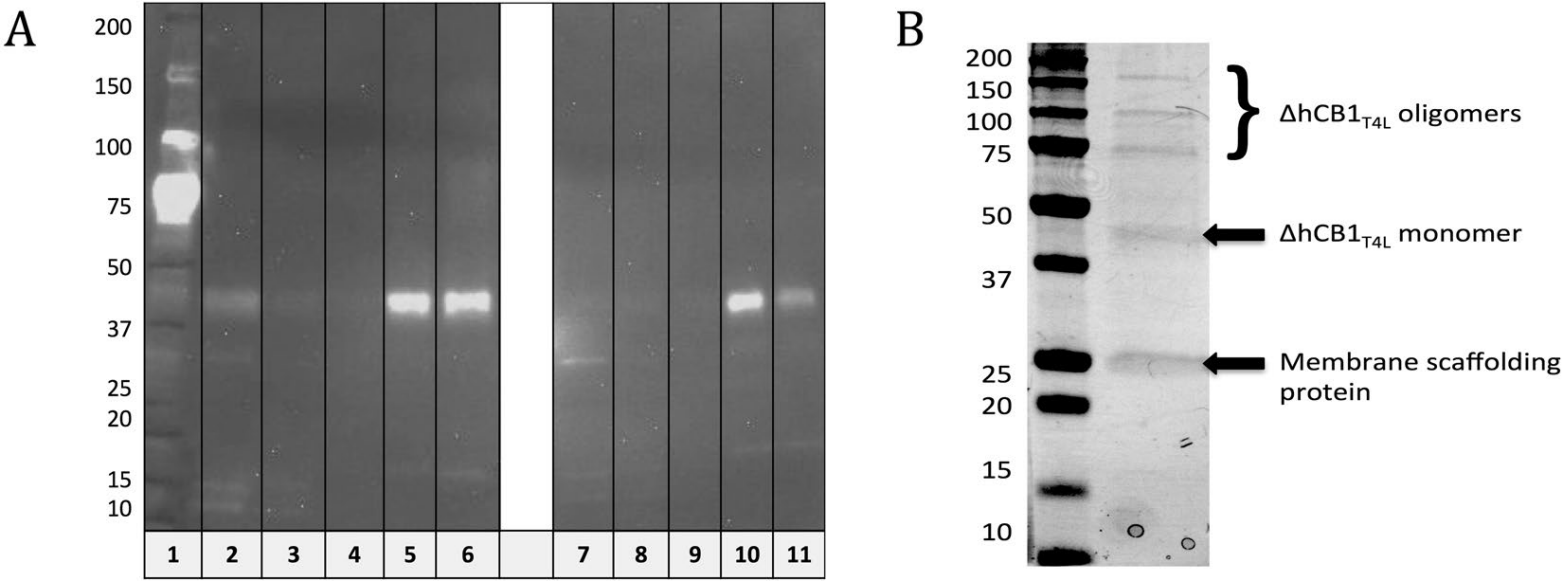
(**A**) Anti-his western blot analysis of the Flag-tag based and his-tag based nanodics-FlaghΔCB1_T4L_his6 complex purifications: lanes 2 and 7 are the unbound to resins fractions; lanes 3, 4, 8, and 9 are the resins wash fractions; and lanes 5, 6, 10, and 11 are the 1^st^ and 2^nd^ elutions from the Flag-tag and his-tag based purifications, respectively. (**B**) Coomassie-stained SDS-PAGE analysis of the cell-free expressed FlaghΔCB1_T4L_his6 in the Flag-tag based purified elution 1.

IMAC on the TALON resin or the FLAG M2 immunoaffinity chromatography was used for nanodics-FlaghΔCB1_T4L_his6 complex purification. The western blot analysis revealed that the nanodics-FlaghΔCB1_T4L_his6 complex was well purified with either his-tag or Flag-tag based purification method (Fig. 6). The coomassie-stained SDS-PAGE analysis confirmed that the quality and quantity of the Flag-tag based purified hCB1 receptor was suitable for MS analysis.

### Mass spectrometric characterization of the cell-free expressed hCB1

The crystal structures of hCB1 in complex with the inverse agonists AM6538 and taranabant were recently published^7,8^. However, characterization of the ligand binding domains with other functionally diverse ligands remained a major goal in our structure-based drug discovery model. Through our “ligand assisted protein structure (LAPS)” approach, we were able to develop a number of high affinity covalent cannabinergic ligands to obtain structural information about the receptor binding site(s)^36^. The final step in our LAPS approach requires the full proteomic characterization of the purified CB1 receptor, before and after ligand treatment, to identify the ligand-modified amino acid residues. Previously, using a “bottom-up” MS-based proteomics method, we were able to achieve greater than 94% sequence coverage of the purified hCB1 receptor following overexpression in *Sf*21 cells^32^. However, the baculovirus expression system is complicated for producing reproducible receptor samples and is also time consuming.

**Figure 7:**
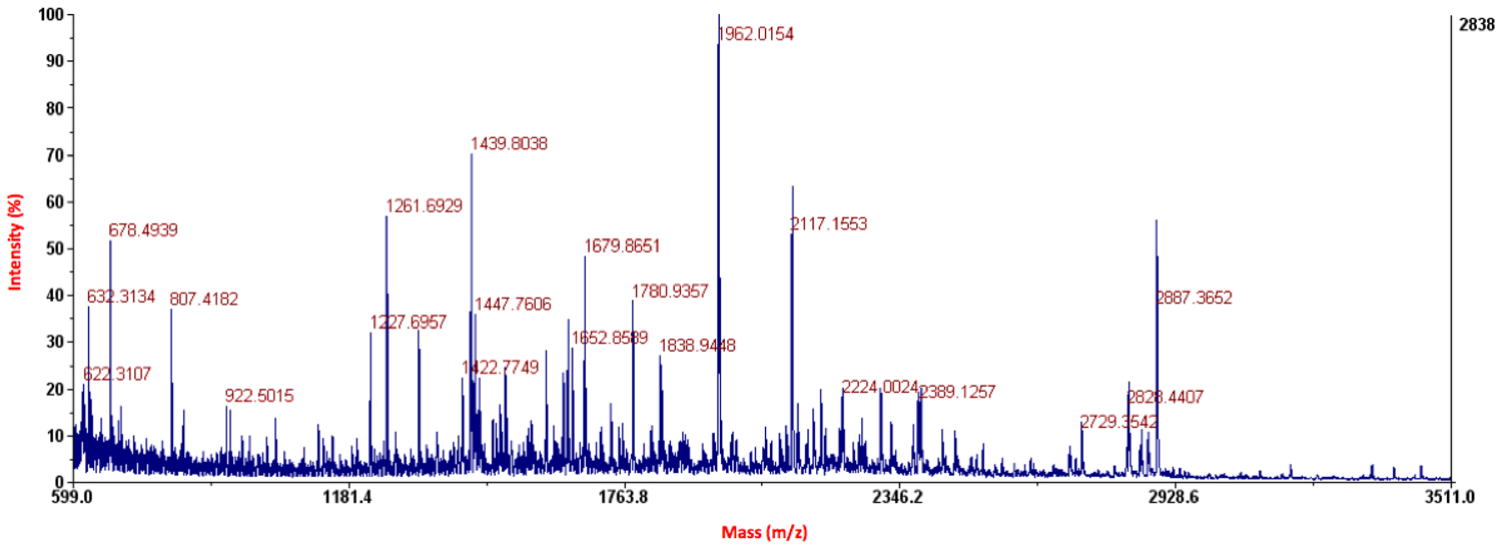
Representative MALDI-TOF reflectron mode MS spectrum of the trypsin digested purified FlaghΔCB1_T4L_his6 receptor.

Here, to ascertain potential use of the cell-free expression system in the first and very important step for the LAPS studies, we expressed the stabilized hΔCB1_T4L_his6 receptor variants *in vitro*, confirmed their ability to bind the CP-55,940 ligand and purified the proteins with either IMAC or immunoaffinity chromatography. The samples for proteomic MS analysis were prepared from the purified shΔCB1_T4L_his6 and FlaghΔCB1_T4L_his6 receptors, followed by reduction with DTT, alkylation with iodoacetamide, desalting on Biospin column and trypsin digestion. MALDI-TOF analysis of the trypsin-digested shΔCB1_T4L_his6 and FlagΔhCB1_T4L_his6 proteins was performed in both reflectron and linear modes (Fig. 7). The peptides identified by MS analysis from the shΔCB1_T4L_his6 and FlaghΔCB1_T4L_his6 samples are listed in Table 1. Overall, we observed ~80% sequence coverage of the *in vitro* expressed and purified hΔCB1his6 receptor using the MALDI TOF/TOF instrument.

**Table 1:** Peptides identified in tryptic digest of the purified shΔCB1_T4L_his6 and FlaghΔCB1_T4L_his6 samples using MALDI-TOF analysis in linear and reflectron mode.

Observed Mass (Da)	Predicted Mass (Da)	E (ppm)	Peptide Sequence	Pos	Mod	C
4097.925	4098.121	47.8	(R)/CRPSYHFIGSLAVADLLGSVIFVYSFIDFHVFHRK/(D)	47–81	CAM	1
767.507	767.445	−80.7	(R)/NVFLFK/(L)	85–90		0
713.523	713.467	−78.7	(R)/IVTRPK/(A)	125–130		0
1354.768	1354.668	−73.4	(K)/AHSHNIFEMLR/(I)	199–209		0
943.608	943.557	−53.9	(R)/IDEGLRLK/(I)	210–217		1
664.474	664.439	−52.3	(R)/LKIYK/(D)	216–220		1
1780.99	1780.912	−44	(K)/DTEGYYTIGIGHLLTK/(S)	221–236		0
787.456	787.431	−31.9	(K)/SPSLNAAK/(S)	237–244		0
988.587	988.542	−45.3	(K)/SELDKAIGR/(N)	245–253		1
1243.693	1243.712	15	(K)/AIGRNTNGVITK/(D)	250–261		1
2259.241	2259.173	−30.2	(K)/DEAEKLFNQDVDAAVRGILR/(N)	262–281		2
2259.269	2259.173	−42.6	(K)/DEAEKLFNQDVDAAVRGILR/(N)	262–281		2
1688.834	1688.933	58.5	(R)/NAKLKPVYDSLDAVR/(R)	282–296		1
3259.672	3259.638	−10.3	(R)/RAALINMVFQMGETGVAGFT NSLRMLQQK/(R)	297–325	3 MO	2
2427.266	2427.216	−20.6	(R)/AALINMVFQMGETGVAGFTN SLR/(M)	298–320		0
3259.672	3259.638	−10.3	(R)/AALINMVFQMGETGVAGFTN SLRMLQQKR/(W)	298–326	3 MO	2
2160.084	2160.134	23.2	(R)/MLQQKRWDEAAVNLAKSR/(W)	321–338	MO	3
2151.357	2151.157	−92.9	(R)/WYNQTPNRAKRVITTFR/(T)	339–355		3
2532.374	2532.23	−56.8	(R)/VITTFRTGTWDAYDQARMDI R/(L)	350–370	MO	2
2828.725	2828.451	−96.8	(R)/VITTFRTGTWDAYDQARMDI RLAK/(T)	350–373		3
3447.849	3447.958	31.7	(K)/TLVLILVVLIICWGPLLAIM VYDVFGKMNK/(L)	374–403	CAM MO	1
746.526	746.459	−89.3	(K)/MNKLIK/(T)	401–406		1
3372.784	3372.824	11.9	(K)/LIKTVFAFCSMLCLLNSTVN PIIYALRSK/(D)	404–432	2 CAM	2
3695.281	3695.609	88.8	(K)/DLRHAFRSMFPSCEGTAQPL DNSMGDSDCLHK/(H)	433–464	2 CAM MO	2
746.526	746.477	−65.5	(K)/STVKIAK/(V)	482–488		1
2234.203	2234.016	−83.9	(K)/VTMSVSTDTSAEALHHHHHH	489–508		0

Columns: E – the difference between observed and predicted peptide mass (ppm); Pos – the start and end of peptide in the hΔCB1_T4L_ sequence; Mod - modifications of underlined residues in the peptide sequence (CAM – carboxyamidomethylated cysteine; MO - oxidized methionine); C – the number of missed cleavages in the peptide. Transmembrane helix (TMH); intracellular and extracellular loops (ICL and ECL); T4 Lysozyme (T4L).

The majority of the identified tryptic hΔCB1_T4L_ peptides belong to the hydrophilic, extracellular and intracellular regions of the receptor and T4 Lysozyme. The amphipathic transmembrane helices are extremely hydrophobic, therefore their loss during sample preparation and resulting problems with their MALDI MS detection were expected. Indeed, TMH1 and 5 were not detected in any of the analyzed samples, while low intensity peaks were observed for TMH 2, 3, 4, 6 and 7. Optimized LC-MS/MS analytical methods for the trypsin-digested samples might be required to obtain the complete coverage of the *in vitro* expressed hCB1 receptor.

## Conclusion

We were able to evaluate, in cells and cell-free *E. coli* expression, the hCB1 receptor variants that were competent to bind CP-55,940 ligand. Under optimized conditions, both the hΔCB1 and hΔCB1_T4L_ (i.e. stabilized by T4 Lysozyme incorporated into the 3rd ICL) were expressed in *E. coli* cells with a receptor content of ~10 μg/L that was competent to bind the CP-55,940 ligand. However neither IMAC nor FLAG M2 immunoaffinity purification was provided the quantity and quality of proteins suitable for MS analysis. *In vitro* expression of the hΔCB1 and stabilized hΔCB1_T4L_ receptors in the presence of nanodiscs produced soluble proteins at a level of ~100 μg in 1 mL of reaction. However only the stabilized hΔCB1_T4L_ receptor was competent to bind CP-55,940. IMAC or FLAG M2 immunoaffinity chromatography was used for the purification of the nanodics-shΔCB1_T4L_his6 or nanodics-FlaghΔCB1_T4L_his6 complexes, respectively. MALDI TOF MS analysis of the purified hΔCB1_T4L_his6 variants, following trypsin digestion and samples preparation for proteomics mass spectrometric analysis, provided ~80% of the hCB1 receptor sequence coverage. We therefore consider that the incorporation of the cell-free expression system in our LAPS approach would be beneficial for the elucidation of hCB1 receptor ligand binding sites.

## Electronic supplementary material

Supplementary Information
