# Supplementary material for: Expression, Purification and Characterization of the Human Cannabinoid 1 Receptor
Source: Sci Rep. 2018 Feb 13;8:2935. doi: 10.1038/s41598-018-19749-5 (PMC5811539; doi:10.1038/s41598-018-19749-5)
Supplement: Supplementary file 1 — Supplementary Information [file 41598_2018_19749_MOESM1_ESM.doc]

**Expression, Purification and Characterization of the Human Cannabinoid 1 Receptor**

Srikrishnan Mallipeddi^1, 2^, Nikolai Zvonok^1, 2^, Alexandros Makriyannis^1, 2, 3^

*^1^ Department of Pharmaceutical Sciences, Northeastern University, Boston, MA 02115, USA*

*^2^ Center for Drug Discovery, Northeastern University, Boston, MA 02115, USA*

*^3^ Department of Chemistry and Chemical Biology, Northeastern University, Boston, MA 02115, USA*

**Supplementary Information:**

**Supplementary Figure S1.** Anti-His western blot analysis of detergent soluble hΔCB1his6 and shΔCB1his6

**Supplementary Figure S2.** [^3^H]CP-55,940 saturation binding to membrane preparations from *E. coli* cells expressed the hΔCB1his6 (am^N^) and shΔCB1his6 (bm^N^).

**Supplementary Figure S3**. Schematic representation of the overlap extension procedure.

**Supplementary Figure S4.** Anti-His western blot analysis of FlaghΔCB1_T4L_his6 receptor expression in *BL*21(DE3) *E. coli* cells.

**Unedited gels/blots.**

**A B**

**Supplementary Figure S1.** **(A)**. Anti-His western blot analysis of detergent DDM soluble hΔCB1his6 (a) and shΔCB1his6 (b) fractions prepared from *BL*21(DE3) cells after 3, 4, 5 h expression and fresh membrane preparations containing hΔCB1his6 or shΔCB1his6 receptors (am^N^ or bm^N^, respectively). **(B)**. Anti-His western blot analysis of detergent soluble hΔCB1his6 and shΔCB1his6 receptors from stored overnight at -80 ^o^C membrane preparations (am^O^ or bm^O^, respectively) and fresh membrane preparations (am^N^).

A B

**Supplementary Figure S2.** [^3^H] CP-55,940 saturation binding to the hΔCB1his6 and shΔCB1his6 in fresh membrane preparations (am^N^ and bm^N^, respectively). The 6-point binding assay was performed in 96-well GF/B filtration plates. The data was analyzed using GraphPad Prism 5 as detailed above.

**Supplementary Figure S3**. Schematic representation of the overlap extension procedure. PCR amplified T4 Lysozyme DNA (in black) flanked with the CB1 complimentary ends (in green and red) and pET26shΔCB1his6 plasmid DNA template (in blue) were denatured, annealed and extended in multiple cycles using Advantage 2 DNA polymerase resulted in pET26shΔCB1_T4L_his6 construct (in yellow). The original methylated plasmid DNA template was digested with *Dpn*I enzyme and newly formed pET26shΔCB1_T4L_his6 DNA was transformed into XL-1 blue *E. coli* cells.

**Supplementary Figure S4.** Anti-His western blot analysis of FlaghΔCB1_T4L_his6 receptor expression in *BL*21(DE3) *E. coli* cells. Total lysate (T), detergent-solubilized fraction in total lysate (S) and detergent-insoluble fraction in total lysate (P) of *BL*21(DE3) cells expressing FlaghΔCB1_T4L_his6 receptor before (0 hr) and after (3, 6 and 9 hrs) induction with IPTG at 25 °C, respectively.

**Unedited Gels/Blots:**

**Figure 3.** Anti-His western blot (lanes 1-7) analysis of FlaghΔCB1_T4L_his6 receptor affinity purification.

**Figure 3.** Coomassie-stained SDS-PAGE (lanes 7-9) analysis of FlaghΔCB1_T4L_his6 receptor affinity purification.

**Figure 4: A** Anti-His western blot analysis optimization of hΔCB1his6 cell free expression. (Lanes 1-8)

**Figure 4: (B)** Anti-His western blot analysis of the cell-free expression of shΔCB1_T4L_his6 (Lane 2) and FlaghΔCB1_T4L_his6 (Lane 3) receptors.

**Figure 6. (A)** Anti-his western blot analysis of Flag-tag based (Lane 2-9) and his-tag based (Lane 10-15) purification of FlaghΔCB1_T4L_his6**.**

**Figure 6. (B)** Coomassie-stained SDS-PAGE analysis of Eluate (Lane 10) from Flag-tag based purification of cell-free expressed FlaghΔCB1_T4L_his6.

**Supplementary Figure S1. (A)** Anti-His western blot analysis of detergent soluble hΔCB1his6 and shΔCB1his6.

**Supplementary Figure S1. (B) (Lane 7-10)** Anti-His western blot analysis of detergent soluble hΔCB1his6 and shΔCB1his6.

**Supplementary Figure S4.** Anti-His western blot analysis of FlaghΔCB1_T4L_his6 receptor expression in *BL*21(DE3) *E. coli* cells.
